# Chewing Betel Quid and the Risk of Metabolic Disease, Cardiovascular Disease, and All-Cause Mortality: A Meta-Analysis

**DOI:** 10.1371/journal.pone.0070679

**Published:** 2013-08-05

**Authors:** Tomohide Yamada, Kazuo Hara, Takashi Kadowaki

**Affiliations:** Department of Diabetes and Metabolic Diseases, Graduate School of Medicine, University of Tokyo, Tokyo, Japan; Cardiff University, United Kingdom

## Abstract

**Background:**

Betel nut (Areca nut) is the fruit of the Areca catechu tree. Approximately 700 million individuals regularly chew betel nut (or betel quid) worldwide and it is a known risk factor for oral cancer and esophageal cancer. We performed a meta-analysis to assess the influence of chewing betel quid on metabolic diseases, cardiovascular disease, and all-cause mortality.

**Methodology/Principal Findings:**

We searched Medline, Cochrane Library, Web of Science, and Science Direct for pertinent articles (including the references) published between 1951 and 2013. The adjusted relative risk (RR) and 95% confidence interval were calculated using the random effect model. Sex was used as an independent category for comparison.

**Results:**

Of 580 potentially relevant studies, 17 studies from Asia (5 cohort studies and 12 case-control studies) covering 388,134 subjects (range: 94 to 97,244) were selected. Seven studies (N = 121,585) showed significant dose-response relationships between betel quid consumption and the risk of events. According to pooled analysis, the adjusted RR of betel quid chewers vs. non-chewers was 1.47 (*P*<0.001) for obesity (N = 30,623), 1.51 (*P* = 0.01) for metabolic syndrome (N = 23,291), 1.47 (*P*<0.001) for diabetes (N = 51,412), 1.45 (*P* = 0.06) for hypertension (N = 89,051), 1.2 (*P* = 0.02) for cardiovascular disease (N = 201,488), and 1.21 (*P* = 0.02) for all-cause mortality (N = 179,582).

**Conclusion/Significance:**

Betel quid chewing is associated with an increased risk of metabolic disease, cardiovascular disease, and all-cause mortality. Thus, in addition to preventing oral cancer, stopping betel quid use could be a valuable public health measure for metabolic diseases that are showing a rapid increase in South-East Asia and the Western Pacific.

## Introduction

Obesity has rapidly become a modern epidemic, with one billion people worldwide being either overweight or obese [1]. In particular, abdominal obesity is associated with insulin resistance that often leads to type 2 diabetes mellitus. Insulin resistance, its associated hyperinsulinemia and hyperglycemia, and the cytokines produced by adipose tissue (adipokines) can also provoke vascular endothelial dysfunction, dyslipidemia, hypertension, and vascular inflammation, all of which promote the development of atherosclerotic cardiovascular disease (CVD) [2], [3].

In Asia, the prevalence of obesity, diabetes, and metabolic disease has increased rapidly in recent years, partly as a result of rapid socioeconomic development [4], [5].

Asia already has 60% of the world’s diabetic population and diabetes is increasing more rapidly in Asia than anywhere else [6]. Such metabolic diseases have a crucial influence on public health, since a modest increase in the risk of morbidity and mortality [7] translates into a substantial social burden, so prevention of these diseases is extremely important.

Betel nut (Areca nut) is the fruit of the Areca catechu tree, which grows in Asia, the tropical Pacific region, and parts of east Africa. It is a major ingredient of betel quid (BQ), which generally consists of areca nut, betel leaf, catechu, slaked lime, and sometimes tobacco [8]. Chewing BQ is common in Central Asian, South Asian, and South-east Asian countries, including Bangladesh, China, India, Pakistan, Philippines, Sri Lanka, Taiwan, and Vietnam [9]. In fact, it has been estimated that 700 million individuals (approximately 10% of the world’s population) chew BQ regularly and it is thought to be the fourth most commonly used psychoactive substance in the world [10].

There are four main arecal alkaloids (arecoline, arecaidine, guvacine, and guvacoline) in betel nut, with arecoline being the main component. These alkaloids bind to GABA receptors in the brain to trigger psychoactive effects such as a sensation of alertness and well-being, but also dizziness [11], [12].

The nitrosated compounds that form when these alkaloids are exposed to gastric acid in the presence of nitrates released by oral bacteria are carcinogenic, and are also similar in structure to various nitrosamines that are well known to be diabetogenic [11].

In fact, the WHO International Agency for Research on Cancer Monograph Working Group has reported that chewing BQ is a known risk factor for oral cancer and esophageal cancer [13].

It was recently proposed that there is an association between inflammatory oral conditions and systemic disorders [14]. Numerous studies have shown that chewing BQ is associated with the risk of various systemic diseases (including metabolic disease, cardiovascular disease, and all-cause mortality), as well as oral diseases, and have generally identified a positive association, although its magnitude has varied [15]–[31].

Thus, clarifying the relationship between chewing BQ and metabolic disease may be important for the development of preventive strategies. Accordingly, we performed a meta-analysis to confirm the influence of chewing BQ on metabolic disease, cardiovascular disease, and all-cause mortality.

## Methods

### Searches

To identify observational studies that had investigated the association between chewing BQ and metabolic disease, cardiovascular disease, and/or all-cause mortality, the electronic databases of Medline, Cochrane Library, Web of Science, and Science Direct were searched from January 1, 1951 until January 30, 2013 using the following key words: (areca nut OR betel nut OR betel quid) AND (mortality OR hypertension OR metabolic OR diabetes OR obesity OR dyslipidemia OR coronary OR heart OR cardiovascular disease). Reference lists of the articles thus identified were also reviewed.

### Selection

Initial screening was based on study titles or abstracts, while subsequent detailed screening employed full-text review. Cohort studies, case-control studies, and cross-sectional studies that assessed the relation between chewing BQ and metabolic disease (obesity, metabolic syndrome, diabetes, hypertension, and dyslipidemia), cardiovascular disease, and all-cause mortality were eligible for inclusion if the following criteria were met: 1) the full text of the report was published in English; 2) the influence of chewing BQ on the relative risk (risk ratio, hazard ratio, or odds ratio) of events was reported with confidence intervals; and 3) the definitions of events were reported.

### Assessment of Validity

To assess the validity of the studies thus identified, each report was appraised with reference to the STROBE statement (an established checklist of items that should be included in articles reporting observational research comprising several study designs and many topic areas) [32]. The Newcastle-Ottawa Scale for assessing the quality of nonrandomized studies in meta-analyses was also used to quantify the validity of each study. This scale is employed to judge a study in three broad areas: selection of the study groups, comparability of the groups, and the method of ascertaining either the exposure or outcome of interest for case-control or cohort studies, respectively [33].

### Data Extraction

Two independent investigators (T.Y. and K.H.) reviewed each study to determine its eligibility and then extracted and tabulated all of the relevant data. Disagreement was resolved by consensus between the two investigators.

The following information was obtained from each study: first author, year of publication, type of study, country, exposure, events, follow-up period, number of subjects, adjustment factors, relative risk, and definitions of events.

### Statistical Analysis

The pooled relative risk (RR) adjusted for possible confounders and its 95% confidence interval (CI) was calculated for each of the events assessed in each study by the DerSimonian-Laird random-effect model weighted with inverse variance. We employed the sex and the ethnic group as independent categories for comparison within each study. In Lin’s study [27], RRs were determined separately for low versus high consumption of BQ. Therefore, we first performed an internal meta-analysis to combine the RR data and then used the combined values for the main meta-analysis. If a study included RRs for both current BQ chewers and ex-chewers, we used the data for current chewers.

We also carried out additional analyses of the studies performed in men with respect to obesity, metabolic syndrome, cardiovascular disease, and all-cause mortality.

Cochrane’s *χ^2^* test and the *I^2^* test were used to evaluate heterogeneity among the studies [34].

Publication bias was evaluated by creating a funnel plot of the effect size versus standard error (SE) for each study, and funnel plot asymmetry was assessed by Begg’s test and Egger’s test. All statistical analyses were performed with Stata 12.0 software (StataCorp, College Station, TX). Results are expressed as the mean with 95%CI, unless otherwise indicated. A *P* value of less than 0.05 was considered significant. All procedures were performed in accordance with the guideline for the meta-analysis of observational studies in epidemiology [35] and the PRISMA statement [36] ***(Checklist S1)***.

## Results

### Search Results

**Figure 1:**
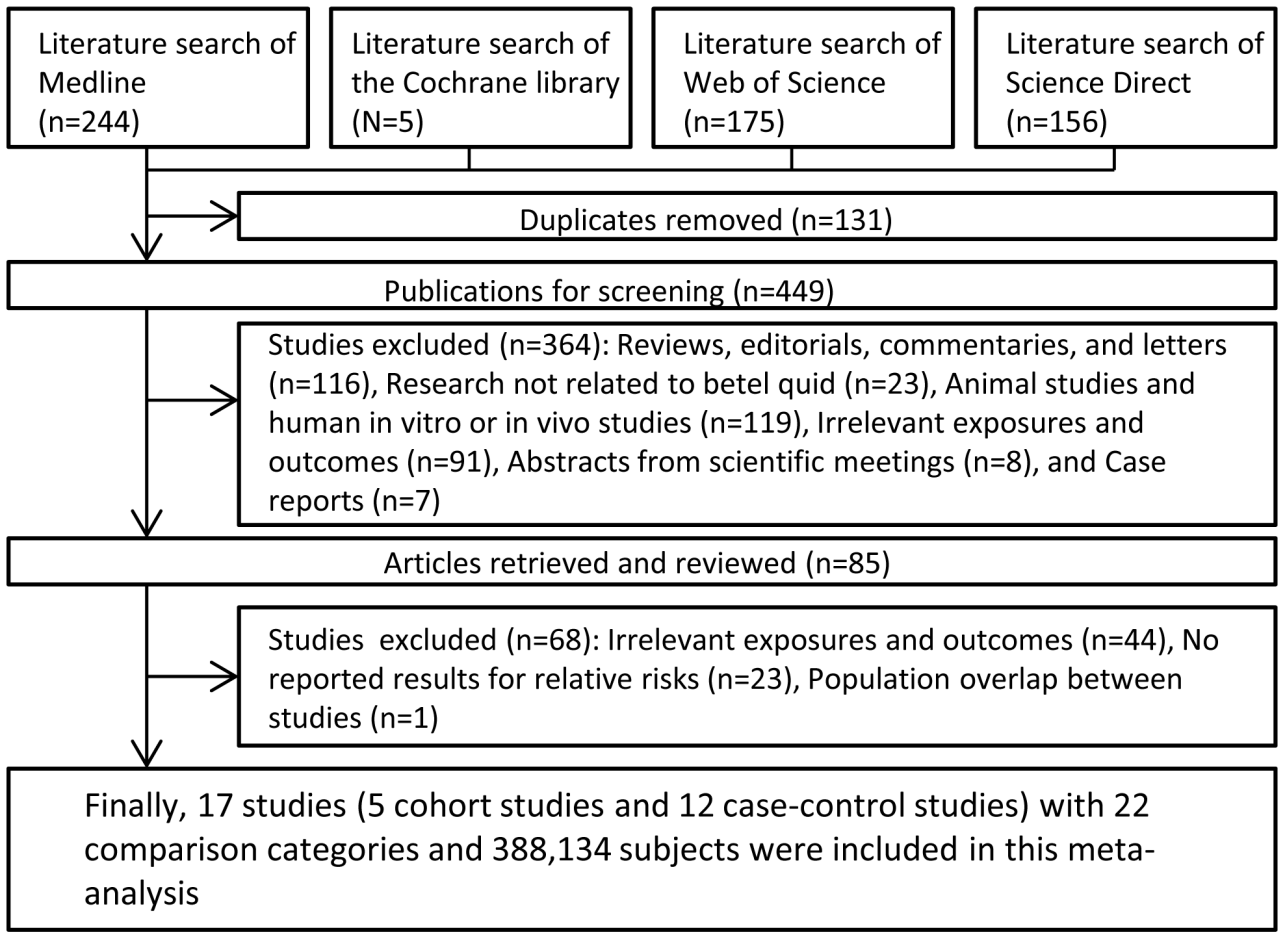
Flow diagram of study selection.

***Figure 1*** shows a flow chart of the study selection process. We identified a total of 580 reports by the database searches, among which 495 reports were excluded after review of the title and abstract, leaving 85 studies for further evaluation. Sixty-eight of these 85 studies were excluded after full-text evaluation, chiefly because of the lack of pertinent data. The remaining 17 studies (5 cohort studies and 12 cross-sectional studies covering 388,134 subjects with 22 comparison categories) [15]–[31] conformed to the selection criteria and were used in this meta-analysis.

### Study Characteristics

***Table 1*** lists the characteristics of the studies. There were 7 comparison categories in the reports about obesity [21], [24]–[27], as well as 4 categories for metabolic syndrome [29]–[31], 4 categories for hypertension [20], [21], 2 categories for diabetes [22], [23], 3 categories for dyslipidemia [28], [30], [31], 6 categories for cardiovascular disease [15]–[19], and 5 categories for all-cause mortality [15]–[18].

**Table 1:** Summary of studies evaluating the association of chewing betel quid with metabolic disease, cardiovascular disease, and all-cause mortality.

Author, Year, Country	Study type	Exposure	Event and Relative risk (95% CI)	Follow-up (yrs)	Number of subjects
Gupta 2005, India (15)^a^	Cohort	Betel quid(areca nut,mishri)	All-cause mortality: (men) 1.1 (0.98–1.24), (women)0.96 (0.83–1.11) CVD: (men) 0.94 (0.82–1.09), (women)1.19 (1.02–1.38)	5.5	97,244 (men and women)
Wen 2005, China (Taiwan) (16)^b^	Cohort	Betel quid	All-cause mortality: (men) 1.5 (1.3–1.7) CVD: (men)1.1 (0.8–1.6)	12.1	19,719 men
Lan 2007, China (Taiwan)(17)^c^	Cohort	Betel quid	All-cause mortality: 1.19 (1.05–1.35)* CVD:1.41 (1.12–1.77)	9.5	6,503 (3,577 men and 2,926 women)
Lin 2008, China (Taiwan)(18)^d^	Cohort	Betel quid	All-cause mortality: 1.4 (1.16–1.7)* CVD: 2.02 (1.31–3.13)*	8.0	56,116 men
Yen 2008, China (Taiwan) (19)^e^	Cohort	Betel quid	CVD: 1.15 (0.99–1.33)*	2.72	21,906 men
Tseng 2008, China (Taiwan) (20)^f^	Case-control	Betel quid	Hypertension: (men) 1.07 (1.01–1.13), (women)1.90 (1.53–2.35)	–	81,226 (37,226 men and 44,000 women with type 2 diabetes)
Heck 2012, Bangladesh (21)^g^	Case-control	Betel quid	Hypertension: (men) 1.36 (0.73–2.53), (women)1.67 (1.08–2.60) Obesity: (men) 0.5 (0.15–1.67),(women) 0.63 (0.3–1.34)	–	7,785 men and women
Tung 2004, China (Taiwan) (22)^h^	Case-control	Betel quid	Type 2 diabetes: 1.29 (1.04–1.60)*	–	14,186 men
Tseng 2010, China (Taiwan) (23)^i^	Case-control	Betel quid	Type 2 diabetes: 1.6 (1.50–1.71)	–	37,226 men
Chang 2006, China (Taiwan) (24)^j^	Case-control	Betel quid	Obesity: 1.48 (1.2–1.81)	–	6,126 men
Lin 2006, China (Taiwan)(25)^k^	Case-control	Betel quid	Obesity: (Aborigine) 1.61 (1.40–1.85)	–	7,144 (3,824 men and 3,320 women)
Ho 2007, China (Taiwan)(26)^l^	Case-control	Betel quid	Obesity: (Aborigines) 1.09 (0.53–2.25).(Non-aborigines) 1.55 (1.17–2.06)	–	8,519 (4,326 men and 4,193 women)
Lin 2009, China (Taiwan)(27)^m^	Case-control	Betel quid	Obesity: 1.89 (1.31–2.72)*	–	1,049 men
Hsu 2010, China (Taiwan) (28)^n^	Case-control	Betel quid	Hypertriglyceridemia: 18.4 (1.19–283) Low HDL cholesterolemia: 5.4 (0.21–35.6)	–	94 (52 men and 42 women)
Chung 2006, China (Taiwan) (29)^o^	Case-control	Betel quid	Metabolic syndrome: (Aboriginal men) 1.92 (1.15–3.27), (Aboriginal women) 1.6 (1.03–2.5)	–	1,466 (men and women)
Guh 2006, China (Taiwan) (30)^p^	Case-control	Betel quid	Metabolic syndrome: 1.06 (0.89–1.30)* Hypertriglyceridemia: 1.2 (0.9–1.5) Low HDL cholesterolemia: 0.86 (0.66–1.1)	–	1,986 (920 men and 1,066 women)
Yen 2006, China (Taiwan) (31)^q^	Case-control	Betel quid	Metabolic syndrome: 1.78 (1.53–2.08)* Hypertriglyceridemia: 1.9 (1.66–2.19) Low HDL cholesterolemia: 0.95 (0.78–1.15)	–	19,839 men

CVD, cardiovascular disease; CI, confidence interval;

a Adjusted for age and education.

b Adjusted for age, alcohol, and education.

c Adjusted for gender, age, living area, hypertension, anemia, heart disease, liver disease, arthritis, physical difficulty, smoking, and alcohol.

d Adjusted for age, BMI, diabetes, hypertension, cholesterol, triglycerides, alcohol, smoking, physical activity, income, and education.

e Adjusted for age, education, occupation, smoking, alcohol, intake of fish, milk, coffee, physical activity, and family history.

f Adjusted for age, diabetes duration, BMI, and smoking.

g Adjusted for age, tobacco smoking (pack-years), BMI at baseline, use of antihypertensive medications at follow-up, education, land ownership, religion, marital status, and daily intake of meat, vegetables, and fruit.

h Adjusted for age, obesity, hypertension, physical activity, education, occupation, total cholesterol, triglycerides, creatinine, uric acid, BMI, and log-transformed BUN.

i Unadjusted.

j Adjusted for hypertension, diabetes, exercise, sedentary job, alcohol, and smoking.

k Adjusted for sex, age, education, marital status, ethnicity, alcohol consumption, and smoking.

l Adjusted for sex, age, marital status, education, income, alcohol (frequency of intake), smoking, and physical activity.

m Adjusted for age, diabetes, hypertension, total cholesterol, high-density lipoprotein cholesterol, triacylglycerol, smoking, alcohol drinking, physical activity, income, and educational level.

n Adjusted for age, sex, and BMI.

o Adjusted for age, educational level, socioeconomic level, exercise, drinking, and smoking status.

p Adjusted for sex, age, smoking, alcohol drinking, dietary intake, and physical activity.

q Adjusted for age, education, physical activity, occupation, smoking habits, alcohol intake, dietary factors, and family history of diabetes, hypertension, and cerebrovascular and cardiovascular disease in second-degree relatives.

* Significant dose-response relationship between chewing betel quid and the event.

Although Guh et al. [30] and Yen et al. [31] described the risks for high blood pressure (systolic blood pressure)≥130 mmHg and/or diastolic blood pressure≥85 mmHg) and hyperglycemia (fasting plasma glucose≥100 mg/dl), these criteria differed from those used to define hypertension and diabetes. Accordingly, we excluded their data from the present meta-analysis.

All of the studies were performed in Asia and were published between 2004 and 2012. Fifteen studies [16]–[20], [22]–[31] were conducted in China (including Taiwan), while 1 was done in the India [15] and 1 was performed in Bangladesh [21]. The size of the study population ranged from 94 to 97,244 subjects (mean: 22,831 subjects). The mean follow-up period ranged from 2.72 to 12.1 years.

### Study Quality

Study quality scores are shown in ***Table S1***. Most of the studies achieved high scores (at least 7 out of 9 points) for quality. The Newcastle Ottawa score was 5 for one study, 6 for three studies, 7 for seven studies, 8 for three studies, and 9 for three studies. Some case-control studies were not allocated a score because a questionnaire or interview was used without blinding to evaluate BQ chewing. In most of the case-control studies, non-response rates were not reported separately for each group.

### Quantitative Data Synthesis (Meta-analysis)

#### 1. Obesity

A total of 5 studies reported on obesity using 7 categories for comparison [21], [24]–[27]. All 5 were case-control studies. Four studies [24]–[27] were performed in China (including Taiwan) and one was done in Bangladesh [21]. These studies included 30,623 subjects. Three studies were limited to men [21], [24], [27]. The definition of obesity varied, since it was defined as a BMI≥27 kg/m^2^ in two studies [24], [26] and as a BMI≥25 kg/m^2^ in two studies [21], [27]. Lin [25] only analyzed Taiwanese aborigines, while Ho [26] analyzed Taiwanese aborigines and non-aborigines separately. Lin [27] found a significant dose-response relationship between BQ consumption and obesity.

**Figure 2:**
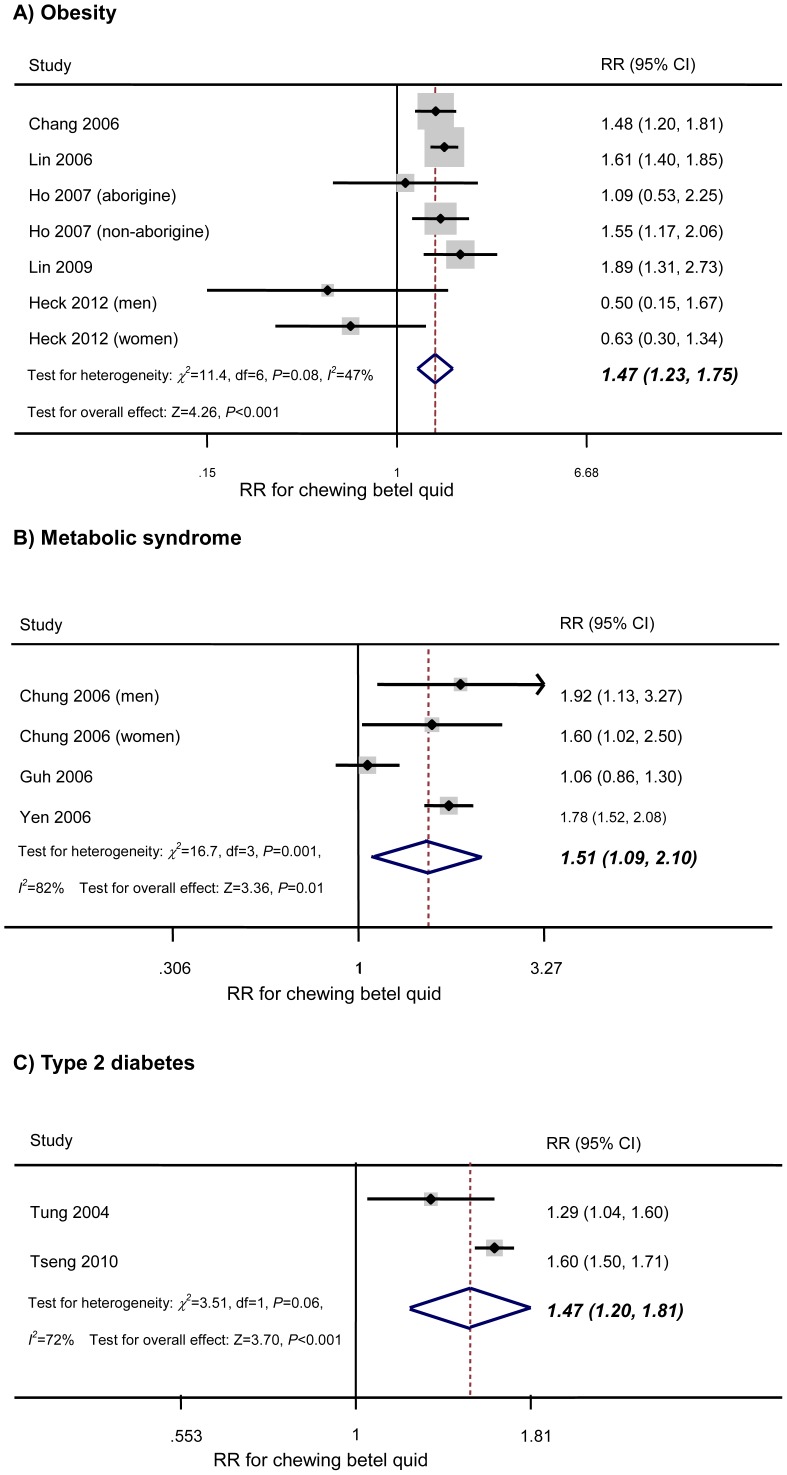
Association of chewing betel quid with obesity, metabolic syndrome, and type 2 diabetes. Forest plots show the association between chewing betel quid and the risk of obesity, metabolic syndrome, or type 2 diabetes. CI = confidence interval; RR = relative risk.

***Figure 2*** ***-A*** shows the results obtained with the random effects model by combining the RRs for obesity. Across the 7 comparison categories [21], [24]–[27], the adjusted RR for BQ chewers compared with non-chewers was 1.47 (95% CI: 1.23 to 1.75; *P*<0.001; *I^2^* = 47%; P for heterogeneity = 0.08) with non-significant heterogeneity. Analysis restricted to studies of men [21], [24], [27] showed a similar RR of 1.49 (95% CI: 1.03 to 2.15; *P* = 0.04). Analysis of only the studies performed in Taiwan [24]–[27] gave an RR of 1.58 (95% CI: 1.42 to 1.75; *P*<0.001) along with a decrease of heterogeneity (*I^2^* = 0%; *P* for heterogeneity = 0.66). Analysis restricted to aborigines [25], [26] revealed an RR of 1.57 (95% CI: 1.3 to 1.9; *P*<0.001; *I^2^* = 7%; *P* for heterogeneity = 0.3).

#### 2. Metabolic syndrome

Three studies [29]–[31] reported on metabolic syndrome using 4 comparison categories. All four studies were performed in China (Taiwan) and there were 23,291 subjects. Chung [29] only investigated aborigines. In all three studies, metabolic syndrome was defined according to National Cholesterol Education Program Adult Treatment Panel III criteria [37]. Two of the three studies [30], [31] identified a significant dose-response relationship between BQ consumption and metabolic syndrome.

Across the 4 comparison categories, the adjusted RR for BQ chewers compared with non-chewers was 1.51 (95% CI: 1.09 to 2.10; P = 0.01; *I^2^* = 82%; P for heterogeneity = 0.001) (***Figure 2*** ***-B***). Analysis restricted to studies of men [29], [31] yielded a similar result, with an RR of 1.79 (95% CI: 1.54 to 2.08; *P*<0.001).

#### 3. Type 2 diabetes

There were 2 case-control studies employing 2 comparison categories that assessed type 2 diabetes [22], [23]. These studies included 51,412 subjects, all of whom were men, and diabetes was diagnosed according to American Diabetes Association criteria [38] in both studies. Tung [22] demonstrated a significant dose-response relationship between BQ consumption and type 2 diabetes.

Across the two studies, the adjusted RR for diabetes in BQ chewers compared with non-chewers was 1.47 (95% CI: 1.2–1.81; *P*<0.001; *I^2^* = 72%; *P* for heterogeneity = 0.06) with non-significant heterogeneity (***Figure 2*** ***-C***).

#### 4. Dyslipidemia

There were 3 case-control studies employing 3 comparison categories that assessed the risk of dyslipidemia (hypertriglyceridemia and low HDL cholesterolemia) related to chewing betel quid [28], [30], [31]. These studies included 21,919 subjects. The adjusted RR for hypertriglyceridemia in BQ chewers compared with non-chewers was significant at 1.64 (95% CI: 1.01 to 2.64; P = 0.04; I2 = 86%; P for heterogeneity = 0.001), but the RR for low HDL cholesterolemia was not significant at 0.94 (95% CI: 0.73 to 1.21; P = 0.62; I2 = 47%; P for heterogeneity = 0.15).

#### 5. Cardiovascular disease

Five studies with 6 comparison categories [15]–[19] and 201,488 subjects included data on cardiovascular disease. All five were cohort studies. The follow-up period ranged from 2.72 years to 12.1 years (mean: 7.6 years). In most studies [15]–[18], cardiovascular disease was defined as a disease of the circulatory system according to the International Classification of Diseases (ICD) death registry system. Lin [18] and Yen [19] found a significant dose-response relationship between BQ consumption and cardiovascular disease.

**Figure 3:**
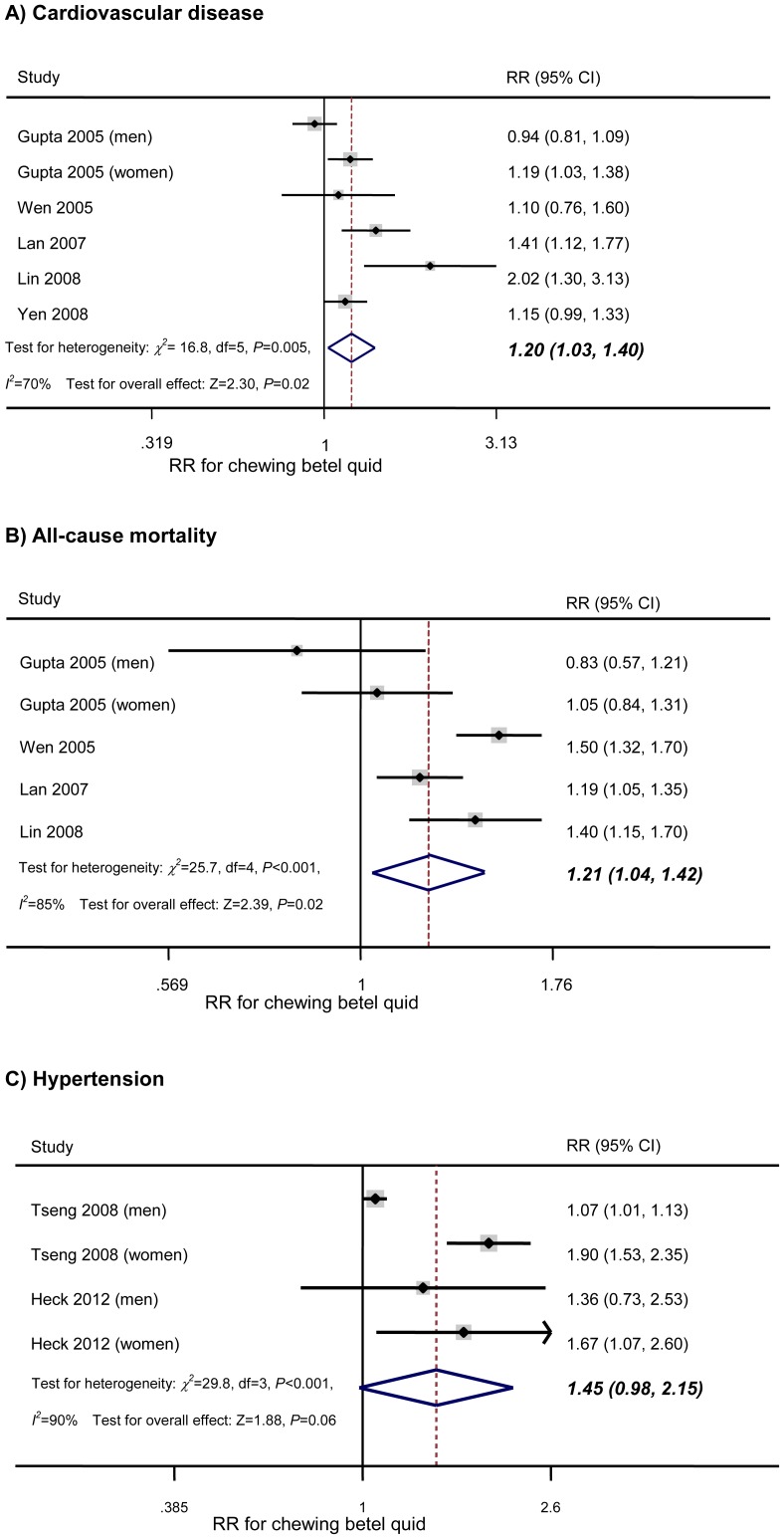
Association of chewing betel quid with cardiovascular disease, all-cause mortality, and hypertension. Forest plots show the association between chewing betel quid and the risk of cardiovascular disease, all-cause mortality, and hypertension. CI = confidence interval; RR = relative risk.

***Figure 3*** ***-A*** displays the results. Across the six comparison categories, the adjusted RR for cardiovascular disease in BQ chewers compared with non-chewers was 1.2 (95% CI: 1.03–1.4; *P* = 0.02; *I^2^* = 70%; *P* for heterogeneity = 0.005). Analysis restricted to studies of men [15], [16], [18], [19] revealed a similar result, with an RR of 1.17 (95% CI: 0.93 to 1.47; *P* = 0.19), but the association was not significant.

#### 6. All-cause mortality

Four studies investigated all-cause mortality using 5 comparison categories [15]–[18]. All four were cohort studies, there were 179,582 subjects, and the follow-up period ranged from 5.5 to 12.1 years (mean: 8.8 years). All-cause mortality was confirmed according to the ICD system. Lan [17] and Lin [18] found a significant dose-response relationship between BQ consumption and all-cause mortality.

Across the 5 comparison categories, the adjusted RR for all-cause mortality in BQ chewers compared with non-chewers was 1.21 (95% CI: 1.04–1.42; *P* = 0.02; *I^2^* = 85%; *P* for heterogeneity<0.001). Analysis restricted to studies of men [15], [16], [18] gave a similar result, with an RR of 1.32 (95% CI: 1.07 to 1.63; *P* = 0.01) (***Figure 3*** ***-B***).

#### 7. Hypertension

Two case-control studies with 89,051 subjects assessed hypertension using 4 comparison categories [20], [21]. Tseng [20] only investigated patients with concomitant diabetes. Hypertension was defined as a systolic blood pressure≥140 mmHg and/or diastolic blood pressure≥90 mmHg in both studies.

Across the 2 studies, the adjusted RR for hypertension in BQ chewers compared with non-chewers was 1.45 (95% CI: 0.98 to 2.15; *P* = 0.06; *I^2^* = 90%; *P* for heterogeneity<0.001) (***Figure 3*** ***-C***).

### Publication Bias

The funnel plot, Begg’s test, and Egger’s test were used to evaluate the potential influence of publication bias on the association between BQ chewing and metabolic disease, cardiovascular disease, and all-cause mortality. The funnel plot did not have an asymmetric pattern, and Egger’s test and Begg’s test both revealed no significant publication bias (all *P*≥0.05).

## Discussion

The present meta-analysis of 17 Asian studies demonstrated that chewing BQ is associated with an increased risk of obesity, diabetes, metabolic syndrome, cardiovascular disease, and all-cause mortality. These findings have certain implications for the prevention of metabolic diseases, especially in view of the recent rapid increase in the prevalence of such diseases in South-East Asia and the Western Pacific.

It was recently suggested that there is an association between inflammatory oral conditions and systemic disorders [14]. Several studies have shown that poor periodontal health is associated with conditions such as diabetes, cardiovascular disease, kidney disease, and low birth weight [XPATH ERROR: unknown variable "start2".], [39], .

A previous meta-analysis [41] investigated the relation between chewing various substances with/without tobacco and the risk of cardiovascular disease, but it assessed the role of chewing tobacco in addition to BQ. In addition, a meta-analysis of the overall risk of metabolic diseases (including obesity, metabolic syndrome, hypertension, diabetes, dyslipidemia) and all-cause mortality associated with chewing BQ has not been performed before.

### Link between Chewing BQ and Systemic Disease

The exact mechanism that links chewing BQ with systemic disease remains unclear, although various mechanisms have been proposed. Nitrosated arecal alkaloid derivatives are proven carcinogens that have been demonstrated to induce tumors throughout the foregut in animals, are also associated with an increased risk of tumorigenesis in humans [8]. When administered intravenously, these alkaloids target organs derived from the foregut such as the brain, nasopharynx, oropharynx, stomach, liver, lungs, and pancreas [11]. This means that the effects of chewing BQ are not limited to tissues with which the quid and its components make direct contact (e.g., the oral cavity and esophagus).

Indeed, Boucher et al. [42] have shown that these nitrosated compounds are diabetogenic in CD1 mice, producing type 2 diabetes associated with obesity. The following mechanisms have been proposed to explain the diabetogenic effect and other effects of chewing BQ.

#### 1) Inflammation

Substances found in betel nut increase the release of inflammatory mediators such as prostanoids, interleukin-6, and tumor necrosis factor-α [43], as well as inducing reactive oxygen species and activating nuclear factor-κB [44], which are changes with the potential to cause chronic inflammation. An increase of C-reactive protein is an independent predictor of type 2 diabetes in apparently healthy women, supporting the hypothesis that subclinical inflammation has a role in the pathogenesis of diabetes [45]. In addition, C-reactive protein can be a predictor of adverse cardiac outcomes [46]. Human studies [47] support the concept that periodontal infection can induce a state of low-grade chronic inflammation, and periodontal therapy has been shown to decrease systemic inflammation [48]. It has also been reported that tumor necrosis factor-α induces insulin resistance [49], and systemic inflammation has been identified as a novel predictor of incident diabetes [50], [51].

#### 2) Effects on adipogenesis, lipolysis, and glucose uptake by adipocyte

Using mouse 3T3-L1 preadipocytes, Hsu et al. demonstrated that arecoline from betel nut inhibits adipogenic differentiation, induces adenylyl cyclase-dependent lipolysis, and interferes with insulin-induced glucose uptake. Therefore, arecoline-induced adipocyte dysfunction may lead to the development of insulin resistance and metabolic disease [28].

#### 3) Influence on appetite

It has been reported that arecoline acts as a competitive inhibitor of γ-aminobutyric acid receptors in the brain, cardiovascular system, and pancreas, suggesting that it may promote an increase in appetite or alter insulin secretion [52].

#### 4) Neurological effects

A human study showed that chewing BQ has a central sympathetic effect, resulting in an increase of the heart rate and increased blood flow in the external and common carotid arteries [53]. In addition, chewing BQ has been associated with coronary artery spasm due to the parasympathetic effect of arecoline on vessels with abnormal endothelium. Furthermore, Asthana et al. [54] reported that arecoline is an effective muscarinic agonist, which crosses the blood–brain barrier and binds to gamma aminobutyric acid receptors, thereby inducing a range of parasympathetic effects.

#### 5) Influence on Vitamin D

The East London Bangladeshi study [55] demonstrated a high prevalence of Vitamin D deficiency among nondiabetic subjects ‘at risk’ of diabetes (spot blood glucose level >6.0 mmol/l <2 h postprandially, or >4.6 mmol/l >2 h postprandially on two separate occasions), while severe deficiency was less common in those ‘not-at-risk’. In addition, chewing BQ is associated with increased expression of the gene coding the enzyme involved in catabolism of Vitamin D (calcitriol) and with a reduction of the serum calcitriol concentration, but not the serum 25(OH)D concentration [56]. Thus, chewing BQ may reduce the activity of available vitamin D.

Recently, vitamin D insufficiency has been recognized as a potential risk factor for metabolic syndrome [57], type 2 diabetes [58] and cardiovascular disease [59], which makes the vitamin D status a potential confounder in any study of BQ chewing since low vitamin D levels are common among south Asians wherever they live [57]. Because chewing BQ may enhance the risks associated with inadequate vitamin D status [56], this is a possible mechanism by which a person’s background can increase cardio-metabolic risk, and it has the potential to create a vicious circle that exacerbates risk when both factors are present.

### Strengths and Limitations

The strengths of this meta-analysis were as follows. First, it included several large-scale cohort studies and case-control studies, with a total of 388,134 subjects. The cohort studies had a relatively long follow-up period (range: 2.7–12.1 years; mean: 7.6 years). In most of the studies, analyses were adequately adjusted by using classical coronary risk factors, such as age, gender, smoking, hypertension, dyslipidemia, and body mass index. Furthermore, evaluation of publication bias using the funnel plot, Begg’s test, and Egger’s test revealed no evidence of bias.

This meta-analysis also had some limitations. First, although we searched several databases, we only reviewed English-language reports and this could have led to selection bias. However, we tried to include as many of the pertinent references as much as possible. Second, data from some of the studies showed significant heterogeneity, which may have been at least partly related to differences of epidemiological characteristics (e.g., the frequency and duration of BQ use). Moreover, case-control studies have more potential for bias compared with cohort studies. It has been reported that BQ chewers are more likely to smoke, drink alcohol, and eat spicy foods compared with non-chewers [9], [60]. Such potential confounding factors could also have a role in predisposing BQ chewers to various systemic conditions. However, the relative risk was adjusted by classical coronary risk factors in most of the studies, and additional analyses stratified by sex and race showed a decrease of heterogeneity. Moreover, significant dose-response relationships between BQ consumption and the risk of events were identified in 7 studies [17]–[XPATH ERROR: unknown variable "next".].

Third, most studies were performed in Taiwan and few studies were done in other areas. Accordingly, the association between chewing BQ and lifestyle-related diseases deserves further investigation elsewhere in the world.

Fourth, the role of gender in relation to BQ chewing and systemic disease remains unclear. Although we wanted to analyze studies performed only in women, most of the studies only assessed men or had more male than female participants, so we could not do this analysis because of the small number of female subjects [15], . It seems that chewing BQ is more prevalent among men than women in Taiwan where most of the studies were performed [21]. Therefore, we have not been able to assess the influence of gender in relationship to cardiovascular disease and metabolic disease among BQ chewers. Accordingly, the results obtained by this meta-analysis are more relevant to men than women.

Even with the above-mentioned limitations, these observational studies provide useful evidence regarding the potential influence of chewing BQ on metabolic diseases, and the overall pooled estimates of risk were robust. Based on our findings, not just physicians but also public health experts and governments should be aware of the risks associated with chewing BQ since use of BQ is the 4th most common addictive habit worldwide. The relationship between BQ and lifestyle-related disease should be investigated by performing further studies, including well-designed and controlled cohort studies in various parts of the world.

### Conclusions

In conclusion, chewing BQ is associated with an increased risk of several metabolic diseases, as well as an elevated risk of cardiovascular disease and all-cause mortality. Thus, in addition to prevention of oral cancer, stopping BQ use could be beneficial with respect to metabolic diseases, which may be especially important in view of the rapidly increasing prevalence of such diseases in South-East Asia and the Western Pacific.

## Supporting Information

Table S1 **Newcastle-Ottawa quality assessment scale for observational studies.**
(DOCX)

Checklist S1 **PRISMA Checklist.**
(DOCX)
