# Supplementary material for: Chewing Betel Quid and the Risk of Metabolic Disease, Cardiovascular Disease, and All-Cause Mortality: A Meta-Analysis
Source: PLoS One. 2013 Aug 5;8(8):e70679. doi: 10.1371/journal.pone.0070679 (PMC3734295; doi:10.1371/journal.pone.0070679)
Supplement: Table S1 — Newcastle-Ottawa quality assessment scale for observational studies. (DOCX) [file pone.0070679.s001.docx]

Supplementary Table 2. Newcastle-Ottawa quality assessment scale for observational studies

| Study reference (author, year) | Selection | Comparability | Outcome/Exposure |
| --- | --- | --- | --- |
| Gupta, 2005 (15) | **** | ** | *** |
| Wen, 2005 (16) | **** | ** | *** |
| Lan, 2007 (17) | **** | ** | *** |
| Lin, 2008 (18) | *** | ** | *** |
| Yen, 2008 (19) | **** | ** | ** |
| Tseng, 2008 (20) | **** | ** | * |
| Heck, 2012 (21) | **** | ** | * |
| Tung, 2004 (22) | **** | ** | ** |
| Tseng, 2010 (23) | *** | * | * |
| Chang, 2006 (24) | **** | ** | * |
| Lin, 2006 (25) | *** | ** | * |
| Ho, 2007 (26) | **** | ** | * |
| Lin, 2009 (27) | **** | ** | * |
| Hsu, 2010 (28) | *** | ** | * |
| Chung, 2006 (29) | *** | ** | * |
| Guh, 2006 (30) | **** | ** | * |
| Yen, 2006 (31) | **** | ** | * |
